# Chaotic light at mid-infrared wavelength

**DOI:** 10.1038/lsa.2016.88

**Published:** 2016-06-17

**Authors:** Louise Jumpertz, Kevin Schires, Mathieu Carras, Marc Sciamanna, Frédéric Grillot

**Affiliations:** 1 CNRS LTCI, Télécom ParisTech, Université Paris Saclay, 75634 Paris Cedex 13, France; 2 mirSense, 86 rue de Paris, bat. Erable, 91400 Orsay, France; 3 LMOPS (Laboratoire Matériaux Optiques, Photonique et Systèmes), CentraleSupélec, Université Paris Saclay, 57070 Metz, France; 4 LMOPS (Laboratoire Matériaux Optiques, Photonique et Systèmes), CentraleSupélec, Université de Lorraine, 57070 Metz, France; 5 Center for High Technology Materials, University of New-Mexico, Albuquerque, 87106-4343 NM, USA

**Keywords:** chaos, nonlinear dynamics, optical feedback, quantum cascade laser

## Abstract

The onset of nonlinear dynamics and chaos is evidenced in a mid-infrared distributed feedback quantum cascade laser both in the temporal and frequency domains. As opposed to the commonly observed route to chaos in semiconductor lasers, which involves undamping of the laser relaxation oscillations, quantum cascade lasers first exhibit regular self-pulsation at the external cavity frequency before entering into a chaotic low-frequency fluctuation regime. The bifurcation sequence, similar to that already observed in class A gas lasers under optical feedback, results from the fast carrier relaxation dynamics occurring in quantum cascade lasers, as confirmed by numerical simulations. Such chaotic behavior can impact various practical applications including spectroscopy, which requires stable single-mode operation. It also allows the development of novel mid-infrared high-power chaotic light sources, thus enabling secure free-space high bit-rate optical communications based on chaos synchronization.

## Introduction

Quantum cascade lasers (QCLs) are unipolar semiconductor lasers offering access to wavelengths from the mid-infrared (IR) to the terahertz domain^1, 2^ and promising impact on various applications such as free-space communications^3, 4, 5^, high-resolution spectroscopy^6^, LIDAR remote sensing^7^ or optical countermeasures. Unlike bipolar semiconductor lasers, stimulated emission in QCLs is obtained via electronic transitions between discrete energy states inside the conduction band^8^. Recent technological progress has led to QCLs operating in pulsed or continuous wave (CW) mode, at room temperature in single- or multi-mode operation, with high powers up to a few watts for mid-IR devices^9, 10, 11^. This spectacular development raises interrogations on the stability of QCLs as little is known on their dynamical properties^12^.

A few theoretical studies have predicted novel types of nonlinear dynamics with enhanced stability for QCLs operating under optical feedback^13, 14^ or injection locking^15, 16^. Over the years, spotty experimental work has shown the possibility of improving QCL features under external control^17, 18, 19, 20, 21^. In particular, optical feedback has demonstrated its potential for noise reduction^22^ or mode selection for widely tunable sources^23^. Very recently, experiments based on optical spectrum measurements have unveiled the existence of five distinct feedback regimes without, however, identifying the complex dynamics dwelling within the QCL^24^.

No resonance feature was found in modulation experiments on QCLs^25, 26^ or in numerical studies^27, 28^, hence contrasting with conventional class B laser diodes and suggesting nonlinear dynamics closer to class A gas lasers^29^. In the last 40 years, a significant amount of work has been performed to understand the physical mechanisms behind nonlinear dynamics or optical chaos in class B interband laser diodes, by applying external forcing mechanisms such as optical injection or feedback^29^. A common feature of these approaches is that relaxation oscillations become undamped when increasing the forcing. As demonstrated in experiments^30^ and simulations^31, 32^ of class A lasers under optical feedback, another scenario exists where the laser is brought into chaos by bifurcating via pulsing dynamics at the frequency of the external cavity. There has however been no evidence so far of similar class A routes to chaos in a semiconductor laser under optical feedback even for quantum dot lasers, which still exhibit class B scenarios despite their highly damped relaxation oscillations^33, 34^.

In this article, we provide the first experimental evidence of a route to chaos in a QCL emitting at mid-IR wavelength. When applying optical feedback with an increasing strength, the QCL dynamics bifurcate to periodic dynamics at the external cavity frequency and later to chaos without an undamping of relaxation oscillations, hence contrasting with the scenarios known in interband laser diodes^29^. Results from a rate equation show very good qualitative agreement with the observed dynamics, and confirm a class A dynamical scenario. In contrast with the spatial (wave) chaos observed earlier in quantum cascade micro-resonators^35^, chaos here does not originate from ray dynamics in a laser cavity but from the temporal nonlinear dynamics driving the evolution of both photon and carrier densities.

## Materials and methods

**Figure 1:**
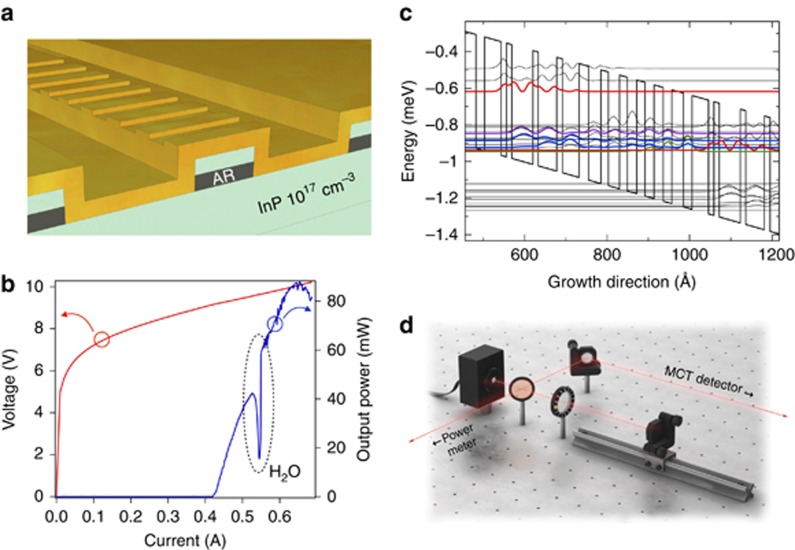
Experiment description. (**a**) Schematic of the device. (**b**) Experimental light (blue) and voltage (red) versus current characteristics of the free-running laser. (**c**) QCL wave functions over one period. In red, upper laser level. In violet, lower laser level. In blue, phonon states. In green, upper injector level. **(d**) Experimental setup. AR, active region.

The lasers under study are distributed-feedback QCLs emitting around 5.62 μm. The active area follows a custom design inspired by Ref. 36 and consists of 30 periods of AlInAs/GaInAs grown by molecular beam epitaxy on a low-doped (10^17^ cm^−3^) InP cladding. The upper InP cladding, grown by metal organic chemical vapor epitaxy, was designed following Ref. 37 to enable single-mode emission using a top metal grating with a coupling efficiency of *κ*≈4 cm^−1^. For the 2-mm-long QCLs used in this article, the obtained *κ*L is close to unity. Furthermore, the width of the QCLs is 9 μm. To improve performances, a high-reflectivity coating on the back facet reduces mirror losses. Figure 1a shows a schematic of the device under study. The laser is processed using a double-trench technology, with SiO_2_ dielectric insulator. For efficient heat extraction, it is episide-down mounted with gold–tin soldering on AlN submount. The AlN submount is then clamped to a copper plate with Peltier thermoelectric cooling. Figure 1b shows the CW light– and voltage–current characteristics of the QCL measured at 10 °C. The threshold of this laser occurs at 425 mA and 9.18 V. The dip in the *L*–*I* curve around 545 mA is a measurement artifact due to the strong water absorption on the path between laser and detector at this wavelength. The electronic structure of one period is presented in Figure 1c. Electrons are injected in the upper laser level from the previous injector by tunneling effect and the radiative transition occurs between the upper and lower levels. The electrons then relax their energy in two phonon states before entering the next injector. Figure 1c also shows the wave functions calculated with a custom heterostructure simulation software named METIS, based on semi-classical Boltzmann equations with thermalized subbands. This software enables calculation of characteristic times with very good agreement between simulation and experiment^38^. For the specific QCL structure under study, the calculated photon and carrier lifetimes are *τ*_p_=4.7 and *τ*_c_=1.3 ps, respectively, resulting in a carrier-to-photon lifetime ratio *T*=0.27, about four orders of magnitude smaller than in interband lasers.

The photon and carrier lifetimes as computed from the simulations bring us to the conclusion that the dynamics of our QCL laser diode will indeed be of class A type. By defining the normalized bias current *P* so that^39^:


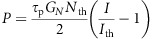


where *G*_*N*_=3.6 × 10^5^ s^−1^ is the differential gain obtained from METIS and *N*_th_=*τ*_c_ *I*_th_/*q* the carrier density at threshold with *q* the electron charge, the eigenvalues that drive the damping of perturbations applied to the laser steady states can be written as:


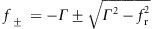


where *f*_r_ is typically called relaxation oscillation frequency in interband lasers, and Γ is the damping rate of relaxation oscillations, expressed as:


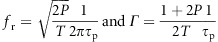


Very close to threshold with *P*=0.02, these parameters take values as high as *f*_r_=13 GHz and *Γ*=419 GHz, the latter being about 30 times higher than the characteristic frequency. In that case, the eigenvalues are real because the term under the square root is positive^26^ and no relaxation oscillations appear in QCLs, as observed in modulation responses^25, 26, 27, 28^.

The experimental setup is depicted in Figure 1d. The laser light is divided into a reflection path and a detection path by a 60/40 beam splitter. In the former, the beam is reflected on a mirror and the feedback ratio is varied using a polarizer and controlled with a power meter, the reflection angle being optimized in order to obtain maximal threshold reduction under optical feedback. With this setup the feedback ratio *f*_ext_, that is, the ratio of the reflected to emitter powers, can reach values up to 25%, and the external cavity length *L*_ext_ can be tuned between 20 cm and 1 m. In the detection path, the light is collected by a Mercury–Cadmium–Telluride photodiode and analyzed with either a 10 gigasample per second real-time oscilloscope or an electrical spectrum analyzer. A mid-IR photodiode with a high bandwidth around 500 MHz was used and the low-cut filter of its preamplifier required the QCL to be operated in pulsed mode. To achieve temperature stability, the pulse length was fixed at 5 μs and a hundred time traces were recorded for each operating point. When computing the statistics of all the time series measured at a given operating point, only a narrow time window towards the end of the pulse was considered. This 5 μs pulse duration was chosen to discard the transient operation appearing at the beginning of the pulse due to heating in the structure. Several pulse lengths were considered, and the transient regime is clearly dominant up to 2 μs. However, a distortion due to the laser driver appears in longer pulses, leading to an optimal pulse duration of 5 μs. Finally, it is important to stress that in contrast with interband structures where the behavior of the laser under optical feedback is dominated by relaxation oscillations, these oscillations do not appear for QCLs within the studied frequency range, as already observed in modulation experiments in QCLs^27, 28^.

## Results and discussion

**Figure 2:**
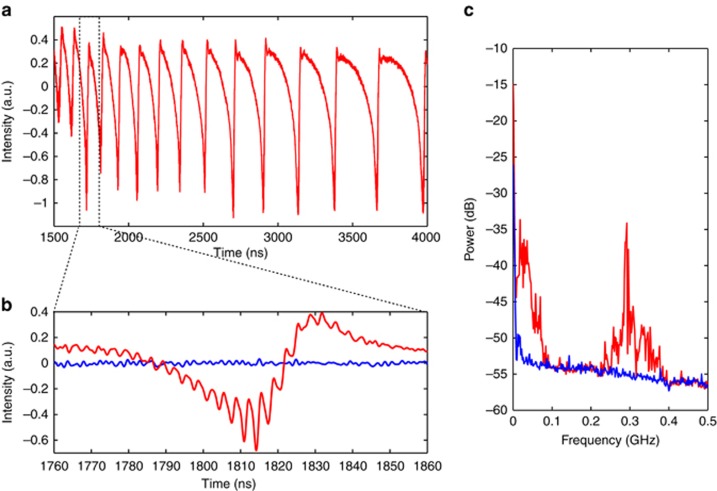
Typical experimental results, for *P*=0.02, *L*_ext_=45 cm and *f*_ext_=3.13%. (**a**) Time trace with slow fluctuations. (**b**) Zoom on one slow period, a faster fluctuation appears at the external cavity frequency (in red), comparison with the free-running case (in blue). (**c**) Electrical spectra that confirm the appearance of the external cavity frequency and of a slower frequency (in red), comparison with the free-running case (in blue).

**Figure 3:**
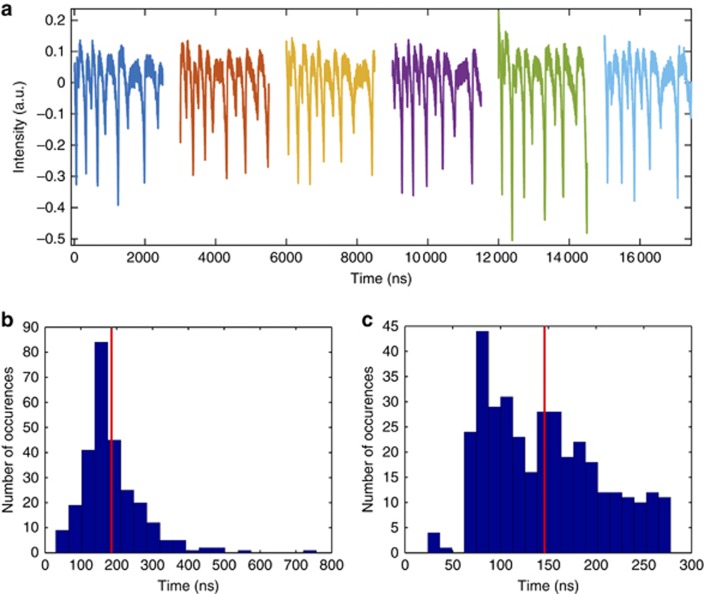
Statistical distribution of the time interval between power drop-outs, for *L*_ext_=35 cm. (**a**) Example of time traces used for the extraction of the statistics, with *P*=0.02 and *f*_ext_=2.70%, considering the last 2.5 μs of several pulses. (**b**) Statistics for *P*=0.02 and *f*_ext_=2.70%. (**c**) Statistics for *P*=0.10 and *f*_ext_=11%, this feedback ratio corresponding to the first occurrence of chaos at this bias current. The red lines indicate the average value.

In Figure 2, time traces and electrical spectra are presented for a normalized bias current *P*=0.02, showing that the QCL under study is sensitive to optical feedback. The operation point was chosen close to threshold to observe the instabilities on a wide range of feedback ratios, while avoiding the water absorption band. However, similar dynamics were observed at higher bias currents. Compared with the free-running case (in blue) where the temporal laser emission remains stable, for moderate feedback ratios (in red) the laser shows erratic pulsing in two distinct frequency ranges. The analysis of the electrical spectra of the QCL subject to optical feedback with varying external cavity length reveals that the fast oscillations occur at the external cavity roundtrip frequency, for instance 0.33 GHz for a 45-cm external cavity as presented in Figure 2c. However, the oscillations at lower frequencies correspond to power drop-outs that resemble low-frequency fluctuations (LFFs), often observed in interband lasers with optical feedback from long external cavities (that is external cavity frequency smaller than the relaxation oscillation frequency)^40, 41^. To further characterize these dynamics, the statistical distribution of the time-interval between two consecutive power drop-outs was studied. The result is presented in Figure 3, for two different bias currents. Figure 3a shows the concatenation of several time traces used for the extraction of the statistics, for a small pump current (*P*=0.02) and feedback parameters equals to *L*_ext_=35 cm and *f*_ext_=2.70%. Each of these time traces corresponds to the last 2.5 μs of the injection current pulses (that is, to remove the transient dynamics, as discussed above). The similarity between the successive time traces suggests that the chaotic trajectories show little sensitivity to initial conditions, hence the chaos is thought to be of low dimension. Under the same experimental conditions, Figure 3b unveils that the shape of the time-interval distribution is a decreasing exponential. Moreover, there exists a range of short time intervals for which there is no occurrence of power dropouts, that is, these are separated by a minimum time span. When increasing the bias current (Figure 3c), a second peak appears at a larger time interval in addition to the exponential decay of the statistical distribution. Furthermore, the minimum as well as the average time interval between two power dropouts are shorter than for the lower current: see in Figure 3 the mean period between two slow oscillations represented by a red line. This statistical distribution and its evolution with the bias current are typical signatures of LFFs, as described in numerous experiments using interband lasers^32, 42, 43, 44^. The identification of LFF dynamics—which is known from interband laser diode studies as deterministic chaos—and the underlying bifurcation sequence from steady state to self-pulsation and further to quasi-periodicity provide evidence of the onset of chaotic light emission in a mid-infrared QCL device.

**Figure 4:**
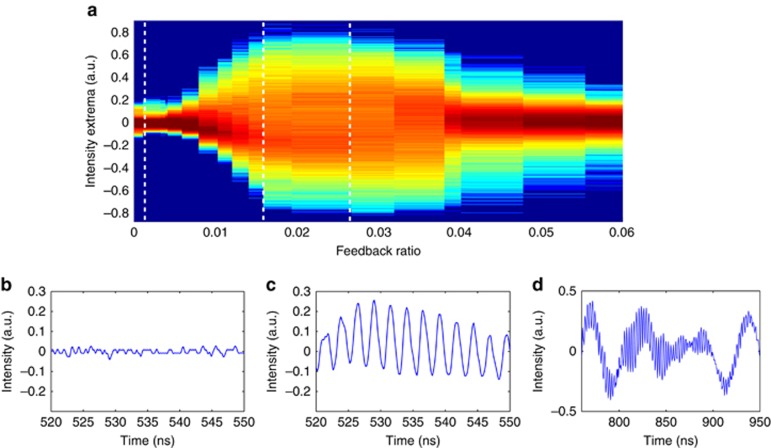
Experimental bifurcation diagram for *P*=0.02 and *L*_ext_=35 cm and associated time series. (**a**) Experimental bifurcation diagram, the white dashed lines correspond to the feedback ratios of the displayed time series. (**b**) Time trace for *f*_ext_=0.11%, showing stable signal. (**c**) Time trace for *f*_ext_=1.58%, showing oscillations at the external cavity frequency. (**d**) Time trace for *f*_ext_=2.66%, showing both LFFs and oscillations at the external cavity frequency.

A more careful sweeping of the feedback ratio at fixed bias current and external cavity length allows recording the bifurcation diagram leading to chaos as shown in Figure 4. The Hopf bifurcation destabilizing the QCL from its steady state is evidenced when increasing the optical feedback strength (Figure 4a). The associated time series recorded before, at and after the Hopf point (Figure 4b–4d, respectively) reveal an unusual route to chaos compared with that typically found in interband lasers. The time-periodic oscillations emerging from the Hopf point (Figure 4c) are indeed regular self-pulsations at the external cavity frequency and not undamped relaxation oscillations as observed close to the first Hopf bifurcation in interband semiconductor lasers with optical feedback. When increasing feedback strength, the QCL output power spreads over a wide range of values corresponding to the onset of an erratic pulsing output in which one recognizes both the fast oscillations at the external-cavity frequency and the slower variations characteristic of LFFs. Note the different timescale in Figure 4d when comparing with Figure 4c. For a further increase of feedback strength the QCL dynamics re-stabilize to a steady state that persists up to the maximum achievable feedback ratio.

Experimentally, when increasing the external cavity length from 25 to 35 to 45 cm at a fixed bias *P*=0.02, the feedback ratio at which the Hopf bifurcation occurs remains constant with an oscillation frequency changing according to the cavity length, whereas the LFF area appears earlier, at 3.18%, 2.66% and 2.40% respectively. This tendency is in qualitative agreement with the cartography previously measured in Ref. 24 by studying the optical spectra, although the values cannot be directly compared due to the different operating points. Furthermore, for a fixed cavity length of 25 cm, the Hopf bifurcation and appearance of LFF occur at 0.59% and 3.18%, respectively, at a bias of *P*=0.02, and it rapidly varies with the pump. For *P*=0.10, these two remarkable points occur at feedback ratios as high as 7.1% and 11.1%, respectively.

To gain insight into the observed bifurcation sequence and confirm the class A dynamical scenario observed experimentally, the behavior of a QCL under optical feedback is studied numerically. The model used for the simulations is the one first introduced by Lang and Kobayashi (LK) for semiconductor lasers subject to optical feedback (LK equations)^45^.


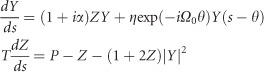


where *Y* represents the slowly varying envelop of the electric field and *Z* the carrier number normalized to the value at threshold. Both equations are normalized to the photon lifetime *τ*_p_. *θ* is the normalized external cavity roundtrip time, Ω_0_ the normalized free-running laser frequency and *η* the normalized feedback coefficient:


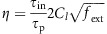


with *τ*_in_ the laser cavity roundtrip time. *C*_*l*_ is the coupling strength coefficient at the front facet, whose expression is complex in distributed-feedback lasers and depends on facet phases as described in Ref. 46. Finally *α* is the linewidth enhancement factor. Also known as linewidth broadening factor or *α*-factor, this parameter quantifies the coupling between phase and amplitude of the electric field in a semiconductor laser^47^. Quantum fluctuations associated with the lasing process affect both the intensity and the phase of the optical field. Although all lasers experience phase fluctuations caused by spontaneous emission, carrier fluctuations in semiconductor lasers provide a second mechanism of phase fluctuations due to the coupling between carrier density, optical gain and refractive index in the optical cavity. This phase–amplitude coupling is a key feature distinguishing semiconductor lasers from any other type of laser, and is responsible for much broader linewidths in such structures. For a mid-infrared QCL, due to the fast carrier lifetime that leads to an almost symmetrical gain, the *α*-factor is thought to be much smaller than in interband semiconductor lasers^48^. However, it is difficult to measure precisely the *α*-factor at the operating conditions. The most common method to quantify the *α*-factor is to extract from measurements of the amplified spontaneous emission spectrum the below-threshold gain and wavelength variations with a change in carrier density^49, 50^. However, studies have shown that the *α*-factor increases significantly above laser threshold^51^. For the QCL under study, as described in a previous work^52^, the α-factor is retrieved from the wavelength shift obtained when increasing the feedback ratio, and is estimated around 1.3, which has also been confirmed from self-mixing experiments^53^. This result is consistent with previous values obtained on CW room temperature mid-infrared QCLs pumped above threshold^48^. Furthermore, both extraction methods are based on optical feedback, using directly the LK equations. The obtained *α*-factor is therefore an effective value that is re-incorporated in the same feedback equations, although it may be slightly higher than the material *α*-factors retrieved close to threshold with other techniques^54^. However, the uncertainty on the measured *α*-factor of 1.3 is high, around 40%, due to the lack of precision on the wavelength measurement induced by the limited resolution of the Fourier-transform IR spectrometer. Therefore this factor was used as a fitting parameter for the simulation, within the measurement uncertainty. The value chosen for the numerical study was thus *α*=1.7.

The *T* value is taken as *T*=0.27 which corresponds to the carrier and photon lifetimes obtained by simulation of the design structure. The values of *P*=0.02 and *θ*=492 correspond to the experimental conditions of the bifurcation diagram presented in Figure 4. The feedback phase Ω_0_ *θ*=−*a*tan (*α*) is adjusted to an arbitrary value. Several noise levels were considered, by adding a less than unity random term multiplied by a noise coefficient (between 10^−14^ and 10^−7^) to both real and imaginary parts of the field at each iteration, without significant modification of the bifurcation diagram. All dimensionless parameters used in the simulations as well as the corresponding experimental values are listed in Table 1.

**Figure 5:**
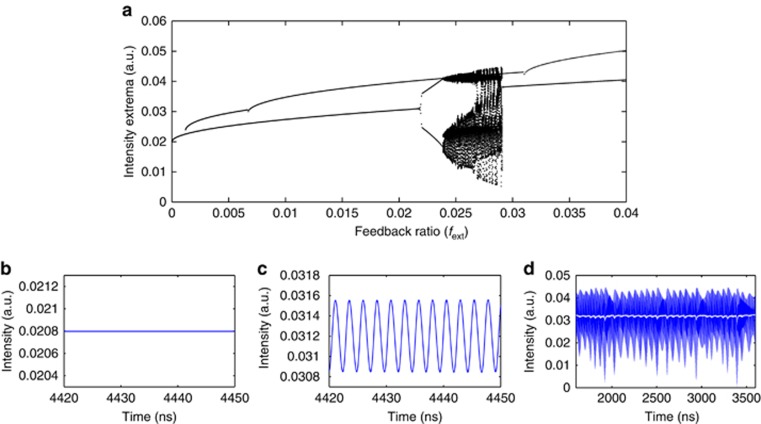
Numerical bifurcation diagram for *P*=0.02 and *L*_ext_=35 cm and associated time series. (**a**) Numerical bifurcation diagram. (**b**) Time trace for *f*_ext_=0.11%, showing stable signal. (**c**) Time trace for *f*_ext_=2.14%, showing oscillations at the external cavity frequency. (**d**) Time trace for *f*_ext_=2.59%, showing both LFFs and oscillations at the external cavity frequency. The white curve corresponds to the filtered trace.

The results of the simulation are presented in Figure 5. For weak feedback the laser emits in a steady-state corresponding to one of the external-cavity mode (ECM) solutions of the LK equations (Figure 5b). The simulated feedback ratio at which the laser emission starts oscillating is *f*_ext_=2.14%, of the same order of magnitude than the experimental one of *f*_ext_=0.59% for the same parameters. Furthermore, as expected from the experiments, this first Hopf bifurcation leads to self-pulsation at the external cavity frequency (Figure 5c) and not to undamped relaxation oscillations. When increasing the feedback strength, the laser dynamics bifurcate to chaos through quasi-periodic oscillations to reach the LFF regime observed in Figure 5d, which is achieved for *f*_ext_=2.69%, a value once again comparable to the corresponding experimental one. Filtering the time trace to remove the high-frequency dynamics better unveils the slow power drop-outs (white curve in Figure 5d). It is interesting to point out that the period between two successive power dropouts is around 250 ns, which is consistent with the experiments.

The bubble of chaotic dynamics corresponding to LFF ends at about *f*_ext_=2.91% and the laser dynamics then settle on a new ECM steady-state with slightly larger output power. When decreasing the feedback strength, the bifurcation diagram shows a cascade of ECM steady-states that coexist with the previous ECM steady-states and therefore also with the periodic and chaotic dynamics. The laser system therefore shows multistability between a large number of either steady or pulsing dynamics when sweeping the feedback strength. The re-stabilization of the dynamics following the onset of chaos has been well observed experimentally in Figure 4. The multistability is however difficult to capture as it requires a fine tuning of the feedback strength. It appears also from the numerical results that the basin of attraction of the LFF dynamics is large and therefore captures most of the system trajectories in phase space.

These results justify the choice of *α*=1.7. Indeed, the Hopf bifurcation and the LFF still appear for lower *α*-factor values, but for much higher feedback ratios, and the chaotic area characterized by LFF is drastically reduced. For instance, in the case *α*=1 the oscillations at the external cavity frequency solely appear around *f*_ext_=75% which is far beyond the maximal feedback ratio achievable experimentally, and the LFF that appear around 80% disappear after a 0.05% increase of the feedback ratio.

The simulated bifurcation scenario of the LK equations confirms a class A dynamical scenario in the studied QCL under external optical feedback. It is interesting to note that while the class A limit of the LK equations has been identified in prior theoretical works in the limit cases corresponding to zero bias current, long delays^31^ with *θ τ*_p_→∞, or high damping^34^, the only experimental observation of a transition to LFF through self-pulsing at the external-cavity frequency and not undamped relaxation oscillations was achieved in gas laser^30^. It is worth mentioning that quantum dot lasers, whose strong damping could theoretically lead to class A scenarios^33, 34^, still behave as class B laser diodes in either optical feedback or optical injection experiments. Here, a class A scenario is reported in the LK model using a different parameter range corresponding to a QCL, and in particular a small value of *T* leading to an absence of relaxation oscillations. Simulations obtained from this class A limit of LK equations shows very good qualitative agreement with experimental results.

## Conclusion

In conclusion, this work reveals experimentally that the output of a mid-infrared semiconductor light source can be rendered chaotic when applying an optical feedback of increasing strength. Owing to the fast carrier relaxation dynamics of QCLs and the absence of relaxation oscillations in such structures, the sequence of bifurcations leading to chaos contrasts with that studied for more than 40 years in interband semiconductor lasers. More specifically the Hopf bifurcation destabilizing the otherwise steady laser output leads to self-pulsations at the external-cavity frequency and not to undamped relaxation oscillations. This dynamical scenario leading to laser chaos is typical for a so-called class A laser, and is here observed for the first time in a semiconductor diode laser under optical feedback.

The onset of chaos in QCLs is of great importance for the current applications of intersubband laser diodes operating in both mid-infrared and terahertz domains. For instance, following the recent development of optical fibers in the mid-IR range^55^, these results suggest that incorporation of mid-IR optical isolators in a butterfly package will be necessary to avoid unwanted optical feedback. Similarly to what occurred for the near-IR systems, the fiber fabrication will also need to be properly controlled in order to limit Rayleigh backscattering^56^. Furthermore, as strong optical feedback in QCLs has proven its efficiency for high power, low threshold, narrow linewidth and single-mode operation for gas spectroscopy, future applications may also consider integrated or monolithic devices for which the feedback parameters would have to be carefully chosen to avoid any chaotic operation. Such monolithic QCLs under stable optical feedback could also provide ultra-low noise mode-locked oscillators at these wavelengths^57^.

On the other hand, chaotic QCLs pave the way to multiple novel mid-IR applications similar to those already existing in the near-IR range, such as chaotic QCL-based LIDAR that would offer jamming-resistant, high-resolution sensing^58^, random bit generation or secured communications based on, for example, chaos modulation for message encryption or synchronized chaos for message transmission^29^. Chaotic QCLs could also be used as broadband mid-IR sources and could be of great interest for optical countermeasures as they offer unpredictable sources.

Further studies will focus on the nonlinear dynamics of various structures of QCLs at different wavelengths under optical feedback and other types of external perturbations.

## Figures and Tables

**Table 1:** Normalized parameters used for simulation and the corresponding experimental parameters

Experimental parameter	Normalized parameter
Carrier lifetime *τ*_c_=1.3 ps	Carrier to photon lifetime ratio *T*=0.27
Photon lifetime *τ*_p_=4.7 ps	
External cavity length *L*_ext_=35 cm	External cavity roundtrip time *θ*=492
Bias current *I*=429 mA	Normalized current *P*=0.02
Free-running wavelength *λ*=5.62 μm	Initial feedback phase Ω_0_ *θ*=−*a*tan (*α*)
*α*=1.3±0.5	*α*=1.7
